# Stable Expression of dmiR-283 in the Brain Promises Positive Effects in Endurance Exercise on Sleep–Wake Behavior in Aging *Drosophila*

**DOI:** 10.3390/ijms24044180

**Published:** 2023-02-20

**Authors:** Qiufang Li, Lingxiao Wang, Yurou Cao, Xiaoya Wang, Chao Tang, Lan Zheng

**Affiliations:** 1 Key Laboratory of Physical Fitness and Exercise Rehabilitation of Hunan Province, Hunan Normal University, Changsha 410012, China; 2 The Center for Heart Development, State Key Laboratory of Development Biology of Freshwater Fish, College of Life Sciences, Hunan Normal University, Changsha 410081, China

**Keywords:** miRNA, aging, sleep–wake behavior, circadian rhythm, exercise

## Abstract

Sleep–wake stability is imbalanced with natural aging, and microRNAs (miRNAs) play important roles in cell proliferation, apoptosis, and aging; however, the biological functions of miRNAs in regulating aging-related sleep–wake behavior remain unexplored. This study varied the expression pattern of dmiR-283 in *Drosophila* and the result showed that the aging decline in sleep–wake behavior was caused by the accumulation of brain dmiR-283 expression, whereas the core clock genes *cwo* and Notch signaling pathway might be suppressed, which regulate the aging process. In addition, to identify exercise intervention programs of *Drosophila* that promote healthy aging, mir-283^SP^/+ and *Pdf* > mir-283^SP^ flies were driven to perform endurance exercise for a duration of 3 weeks starting at 10 and 30 days, respectively. The results showed that exercise starting in youth leads to an enhanced amplitude of sleep–wake rhythms, stable periods, increased activity frequency upon awakening, and the suppression of aging brain dmiR-283 expression in mir-283^SP^/+ middle-aged flies. Conversely, exercise performed when the brain dmiR-283 reached a certain accumulation level showed ineffective or negative effects. In conclusion, the accumulation of dmiR-283 expression in the brain induced an age-dependent decline in sleep–wake behavior. Endurance exercise commencing in youth counteracts the increase in dmiR-283 in the aging brain, which ameliorates the deterioration of sleep–wake behavior during aging.

## 1. Introduction

Disturbances in circadian rhythms and disruptions in the sleep–wake cycle that occur during aging are associated with age-dependent functional deterioration of the nervous system and are important determinants of longevity and a healthy lifespan [1,2]. There is evidence that circadian rhythm disturbances and sleep disorders can lead to premature aging and age-related diseases [3,4,5]. Decreased sleep quality in the elderly is thought to be the result of a reduced ability to initiate and/or maintain sleep, which may be associated with damage to the prefrontal cortex of the brain [2,6] and alterations of sleep–wake regulatory mechanisms in the pons and hypothalamus [2]. However, the molecular mechanisms underlying age-related decreases in sleep–wake behavior are not fully clear.

MicroRNAs (miRNA) are non-coding RNAs involved in post-transcriptional regulation of gene expression, which regulate a variety of biological functions by degrading mRNA expression and/or preventing its translation into proteins. Several studies have demonstrated that miRNAs are regulators of healthy aging and longevity in the organism [7,8,9,10], and play an important role in regulation of the biological clock [11,12]. Further studies have shown that there are significant differences in the expression levels of miRNAs between sleep and wakefulness [13,14], and in patients with sleep disorders [15], although the mechanisms of regulation remain to be fully investigated.

Over the past few decades, *Drosophila melanogaster* has become an excellent model organism for studying the complex interactions between aging and circadian rhythms. *Drosophila*’s circadian rhythm regulation mechanisms and sleep regulation principles are highly consistent with mammals [16] and show common features such as sleep fragmentation, low sleep efficiency, and reduced wakefulness thresholds in older populations [17,18]. Exercise as a beneficial lifestyle has a positive contribution to healthy aging and longevity. In terms of sleep and circadian rhythm, moderate age-matched exercise can improve several aging characteristics [2] and reset the circadian biological clock [19]. Therefore, we performed a study on the miRNA-regulated mechanism of aging decline in circadian rhythms relying on the *Drosophila* model and further explored the ameliorating effect of exercise initiation in early and mid-life on the improvement of sleep–wake behavioral decline.

## 2. Results

### 2.1. Drosophila Exhibits Age-Dependent Decline in Sleep–Wake Behavior during Aging

**Figure 1:**
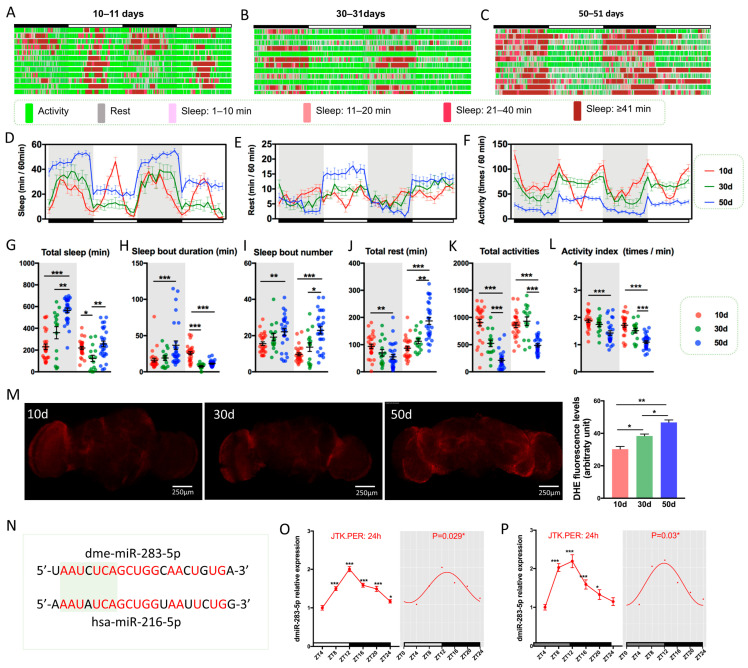
Age-dependent changes in sleep–wake behavior and brain ROS levels in *Drosophila*. (**A**–**C**) Sketches of sleep–wake behavior of young (10–11 days old), middle-aged (30–31 days old), and old (50–51 days old) *Drosophila* for 2 consecutive days. Black bars indicate night-time and white bars indicate daytime. *n* = 25–32. (**D**–**F**) Sleep time per hour, rest time per hour, and activity per hour for young, middle-aged, and old flies for 2 consecutive days, respectively. (**G**–**L**) The sleep–wake changes in young, middle-aged, and old flies at night and during the day, respectively, with the gray part indicating night-time and the white part indicating daytime (Kruskal–Wallis test). Of these, (**G**) indicates total sleep time at night/day, (**H**) indicates sleep duration at night/day, (**I**) indicates the number of sleep segments at night/day, (**J**) indicates total rest time at night/day, (**K**) indicates total activity numbers at night/day, and (**L**) indicates the number of activities per minute during wakefulness at night/day. (**M**) Representative images of ROS levels in young, middle-aged, and old *Drosophila* brains, which were presented with the fluorescent probe of Dihydroethidium (DHE), with 5–10 brains imaged for each condition, the average fluorescence intensity at each age assessed using ImageJ, and entire brain regions labeled as regions of interest (one-way ANOVA test). (**N**) Homology comparison of *Drosophila* dme-miR-283-5p and human hsa-miR-216-5p, with the seed sequence shaded in green. (**O**,**P**) The rhythmic expression of dmiR-283 in young *Drosophila* brains. (**O**) indicates the expression of dmiR-283 in a 10-day-old *Drosophila* brain under 12h:12h LD conditions. (**P**) indicates that after 3 days of LD entrainment after fledge, 3 days of DD rearing was performed and brain dmiR-283 expression was detected at day 7 in age. Total RNA of approximately 30 brains was obtained at 4 h intervals for detection, with the reference gene *U6*, and differential analysis was performed with reference to the expression level of zeitgeber time 4 (ZT4). JTK.PER was used for gene expression period analysis and CircaCompare was used for circadian rhythm significance analysis. ZT0 indicates that lights were turned on, and ZT12 indicates that lights were turned off. * *p* < 0.05, ** *p* < 0.01, *** *p* < 0.001.

Loss of sleep stability is one of the main features of aging, as evidenced by decreased sleep continuity, more fragmented sleep, and increased night-time wakefulness and daytime sleep [20]. In addition, excessive sleep (≥10 h) in the elderly was considered to be an underlying disease or poor health condition [21]. Therefore, we analyzed the sleep–wake pattern and found that 10 days (10d) flies gradually entered the sleep state after the lights were turned off, woke up spontaneously approximately 2 h before the lights were turned on, and “napped” in the middle of the day; their activities were mainly concentrated before and after the lights were turned on/off (Figure 1A), and the sleep–wake cycle was maintained at 12 h (Figure 1D–F, Table S1). With increasing age, 30 days (30d) and 50 days (50d) flies developed increased nocturnal sleep, disappearance of daytime “naps”, increased fragmented sleep (Figure 1B,C,G,H), and a tendency for nocturnal sleep and daytime wakefulness to be light-induced (Figure 1B,C), suggesting a gradual decline in endogenous circadian rhythms in aging *Drosophila*. Circadian rhythm analysis revealed that the sleep–wake cycle shifted to 24 h in 30d and 50d flies (Table S1), and the amplitude and mesor values of sleep and activity showed an age-dependent decrease (Figure 1E,F, Table S1). Additionally, 50d flies noticeably had significantly more resting time and significantly lower autonomous activity behavior during daytime awakenings (Figure 1J,K), indicating an increased “sedentary and less active” state in older flies and a reduced frequency of activity during awakening (Figure 1L), which further suggests that 50d flies are in a disease state or are otherwise dysfunctional [22,23].

We measured aging brain ROS by dye assays and found that ROS levels increased with age (Figure 1M), which is consistent with previously reported oxidative damage [24], indicating that aging *Drosophila* brains accumulate higher levels of superoxide anions. Vaccaro et al. also found significantly increased levels of ROS in *Clk*^AR^ mutant *Drosophila* brains [24], further associating an aging brain with the circadian biological clock.

### 2.2. dmiR-283 Is Rhythmically Expressed in Drosophila Brains

Precursor miRNA and mature miRNA have been reported to display circadian expression patterns and are conserved from plants to mammals [25,26]. The results of in situ hybridization in *Drosophila* embryos suggested that dmiR-283 was expressed in the nervous system and the silencing of dmiR-283 induced neurological defects [27], whereas the expression and functions of dmiR-283 in adult *Drosophila* remain unreported. *Drosophila* dmiR-283-5p has a high homology with human hsa-miR-216a-5p (Figure 1N, 77.27%), and the nucleotides of the seed sequence are also highly congruent (Figure 1N, 85.71%), demonstrating that dmiR-283 has similar regulatory roles to mammalian miR-216a. It has been demonstrated that miR-216a was involved in regulating the progression of Alzheimer’s disease and Parkinson’s disease, which indicates that miR-216a plays an important role in the process of neurodegeneration and provides a new perspective for the study of dmiR-283. To investigate whether there is a circadian rhythm in the expression pattern of dmiR-283, we collected 10d brains at 4 h intervals, and 12 h: 12 h light-dark (LD) entrainment as well as a dark–dark (DD) environment were included. For the mir-283 expression assay, the 5′ end of mature mir-283 was detected, representing the expression of mir-283. The qPCR results showed a significant 24 h expression rhythm of dmiR-283 in both LD and DD situations (*p* < 0.05), with the highest expression at zeitgeber time 12 (ZT12) and reduced expression levels before and after lights on (ZT24) (Figure 1O,P), which indicated that the circadian rhythm changes of sleep–wake behavior in *Drosophila* may be related to the rhythmic expression of dmiR-283.

### 2.3. LNvs-Specific Knockdown of dmiR-283 Induces the Aging Sleep–Wake Behavior

**Figure 2:**
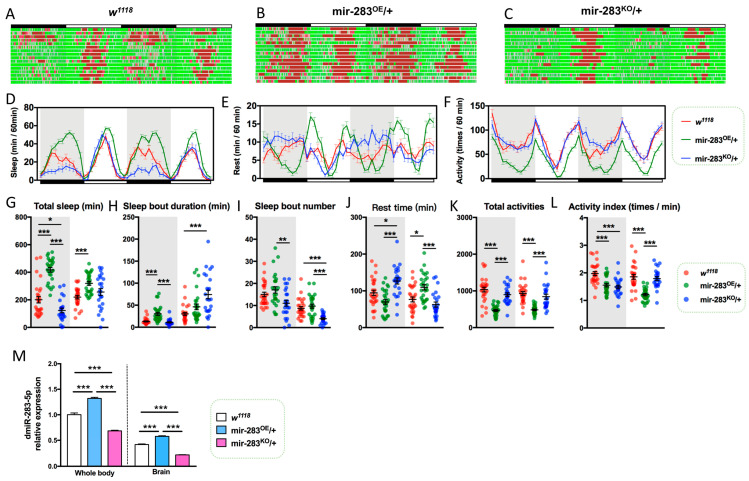
Changes in sleep–wake behavior in dmiR-283 overexpression and knockdown in *Drosophila*. (**A**–**C**) Sketches of sleep–wake behavior of wild-type (*w^1118^*), dmiR-283-overexpressing (mir-283^OE^/+), and dmiR-283 knockdown (mir-283^KO^/+) young flies (10-11 days old) for 2 consecutive days, *n* = 28–32. (**D**–**F**) Sketches of sleep–wake behavior of *w^1118^*, mir-283^OE^/+, and mir-283^KO^/+ young flies for 2 consecutive days of sleep time per hour, rest time per hour, and activity per hour. (**G**–**L**) Total sleep time at night/day (Kruskal–Wallis test) (**G**), sleep duration at night/day (**H**), the number of sleep segments at night/day (**I**), total rest time at night/day (**J**), total activity numbers at night/day (**K**), and the number of activities per minute during wakefulness at night/day (**L**) for *w^1118^*, mir-283^OE^/+, and mir-283^KO^/+ young flies. (**M**) Relative expression levels of dmiR-283-5p in the whole body and brain of *w^1118^*, mir-283^OE^/+, and mir-283^KO^/+ young flies (ZT8), with the reference gene *U6* (one-way ANOVA test). * *p* < 0.05, ** *p* < 0.01, *** *p* < 0.001.

To investigate the association of dmiR-283 with sleep–wake behavior, we analyzed the autonomic activity behavior of 10d *Drosophila* after the overexpression and knockdown of dmiR-283. The results showed that mir-283^OE^/+ flies exhibited increased sleep behavior (Figure 2B,D,G,H), decreased mobility (Figure 2B,F,K), and increased daytime rest time (Figure 2J), which was consistent with the sleep–wake behavior of wild-type 50d flies (Figure 1). In contrast, mir-283^KO^/+ resulted in reduced sleep time and increased wakefulness at night (Figure 2G,J), and a sleep rebound that may be the reason for the prolonged daytime sleep duration (Figure 2H). In addition, both the overexpression and knockdown of dmiR-283 in *Drosophila* reduced activity frequency (Figure 2L), suggesting that the stable expression of dmiR-283 is necessary to maintain normal sleep–wake behavior. CircaCompare analysis revealed that mir-283^OE^/+ *Drosophila* had delayed sleep and activity phases (Table S2) and their resting rhythms exhibited abnormal peaks and troughs (Figure 2E), which indicated that the overexpression of dmiR-283 disrupts the stable expression of endogenous circadian rhythms. The mir-283^OE^/+ and mir-283^KO^/+ brains showed significantly increased and decreased dmiR-283 expression (Figure 2M), further suggesting that disrupted regular dmiR-283 dynamic expression in the brain would lead to abnormal sleep–wake behavior.

**Figure 3:**
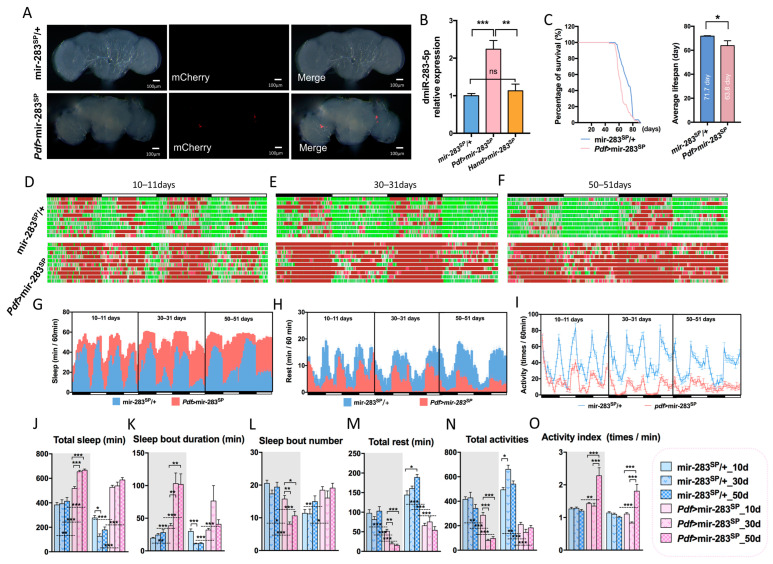
Effects of the LNvs-specific knockdown of dmiR-283 on lifespan and sleep–wake behavior. (**A**) Fluorescence acquisition images of 3-day-old *Drosophila* brains from control (mir-283^SP^/+) and LNvs-specific expression of mir-283 sponge (*Pdf* > mir-283^SP^) groups, with mCherry fluorescence labeled where mir-283 sponge was driven; from left to right, bright field, mCherry filter, merged; *n* = 8–10. (**B**) Expression levels of dmiR-283-5p relative to *U6* in 10-day-old *Drosophila* brains of mir-283^SP^/+, *Pdf* > mir-283^SP^, and *Hand* > mir-283^SP^ (one-way ANOVA test). (**C**) Survival and mean lifespan of mir-283^SP^/+ and *Pdf* > mir-283^SP^ flies, *n* = 80–100 (*t*-test). (**D**–**F**) Sketches of sleep–wake behavior of mir-283^SP^/+ and *Pdf* > mir-283^SP^ flies on 2 consecutive days at youth, middle age, and old age, *n* = 20–32. (**G**–**I**) Sleep time per hour, rest time per hour, and activities per hour for mir-283^SP^/+ and *Pdf* > mir-283^SP^ flies on 2 consecutive days in youth, middle age, and old age, respectively. (**J**–**O**) Total sleep time at night/day (Kruskal–Wallis test) (**J**), sleep duration at night/day (**K**), the number of sleep segments at night/day (**L**), total rest time at night/day (**M**), total activity numbers at night/day (**N**), and the number of activities per minute during wakefulness at night/day (**O**) in mir-283^SP^/+ and *Pdf* > mir-283^SP^ flies at youth, middle age, and old age. * *p* < 0.05, ** *p* < 0.01, *** *p* < 0.001.

Approximately 150 circadian neurons constitute the biological clock network of the *Drosophila* brain [28], where the PDF is secreted by *Drosophila* LNvs, similar in function to the mammalian suprachiasmatic nucleus (SCN), and primarily regulates circadian pacing, sleep, and light-mediated arousal [29]. We knocked down the expression of dmiR-283 by driving the dmir-283 sponge through *Pdf*-Gal4 specifically expressed in LNvs. The mir-283 sponge was fluorescently labeled with mCherry (Figure 3A), which similarly localized the expression of PDF protein while highlighting the LNvs. However, the expression levels of dmiR-283 were significantly increased in whole brains with the PDF-specific knockdown of dmiR-283 (Figure 3B), implying that the LNvs-specific knockdown of dmiR-283 might lead to a compensatory increase in other parts of the brain. To verify this conjecture, we expressed dmir-283 sponge in the heart with *Hand*-Gal4 and detected no significant change in the expression level of brain dmiR-283 (Figure 3B).

Lifespan is considered to be a comprehensive indicator for detecting physiological health in *Drosophila*, and miRNAs have been demonstrated to be involved in regulating neuronal activity and lifespan [30,31,32,33]. Several studies have proven that disruption of the biological clock function, both in insects and rodents, has many detrimental effects on healthy lifespan, including oxidative damage, increased accumulation of protein glycosylation, disruption of metabolic homeostasis, premature aging, and age-related diseases [3,34,35,36,37,38]. Therefore, we counted the survival rate of knockdown dmiR-283 in *Drosophila* LNvs to further clarify whether brain dmiR-283 is involved in lifespan regulation. Statistics on survival displayed an earlier mortality and shorter average lifespan in *Pdf* > mir-283^SP^ flies (Figure 3C), indicating that the stable expression of brain dmiR-283 is also important for maintaining a normal lifespan.

Thus, does the LNvs-specific knockdown of dmiR-283 induce a circadian phenotype of aging? Sleep–wake profiling revealed that *Pdf* > mir-283^SP^ flies had increased daytime and night-time sleep (Figure 3D–F), prolonged sleep duration and total sleep time (Figure 3J,K), and reduced resting time (Figure 3M) and activity behavior (Figure 3N) at 10d, 30d, and 50d compared with the age-matched control flies, which resembled the age-dependent increased sleep phenotype. Furthermore, *Pdf* > mir-283^SP^ flies had significantly lower circadian rhythm amplitude and increased mesor for sleep rhythms, and decreased mesor for rest and activity rhythms (Figure 3G–I, Table S3), indicating that the LNvs-specific knockdown of dmiR-283 induces a senescent circadian rhythm phenotype. In conclusion, our findings suggest that brain dmiR-283 is an important miRNA regulating sleep–wake actions.

### 2.4. Prospective Mechanisms of dmiR-283 to Regulate age-Dependent Circadian Rhythm Decline and Sleep Prolongation

**Figure 4:**
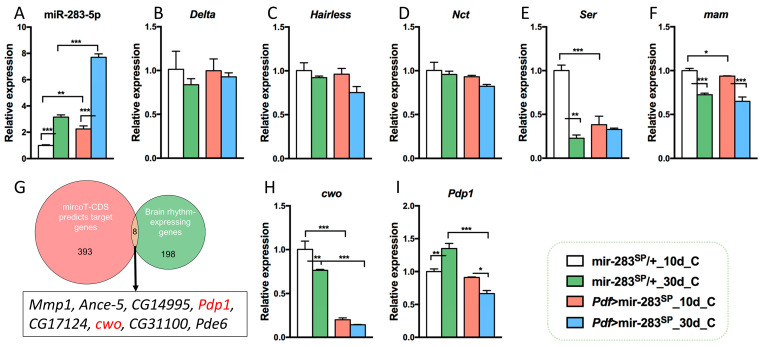
Expression of dmiR-283 and of its predicted target genes in mir-283^SP^/+ and *Pdf* > mir-283^SP^ in young and middle-aged *Drosophila* brains. (**A**) Expression levels of dmiR-283-5p relative to *U6*. (**B**–**F**) dmiR-283-5p predicts target gene enrichment in the Notch signaling pathway, and related gene mRNA expression levels with reference to *rp49*. (**G**–**I**) Intersection analysis of dmiR-283-5p predicted target genes with brain-rhythm-expressing genes (**G**), and relative expression levels of core rhythm genes *cwo* (**H**) and *Pdp1* (**I**) with reference to *rp49*. * *p* < 0.05, ** *p* < 0.01, *** *p* < 0.001 (one-way ANOVA test).

miRNAs perform post-transcriptional regulation by target binding mRNAs; typically, a single miRNA can combine hundreds of target mRNAs [39]. The 401 potential target mRNAs for dmiR-283 were predicted by the DIANA microT-CDS online tool and mainly enriched in the Notch signaling pathway (*Delta*, *Hairless*, *Nct*, *ser*, and *mam*, *p* = 0.0055). Previous studies have shown that the Notch signaling pathway is evolutionarily conserved and plays a regulatory role in the developing and post-developmental nervous system [40], whose over-activation or inhibition leads to drowsiness behavior in *C. elegans* [41]. Therefore, we examined the expression levels of related genes in the brain and found that both aging and LNvs knockdown of dmiR-283 resulted in the significant upregulation of dmiR-283 expression throughout the brain (Figure 4A), while *Ser* and *mam* were downregulated (Figure 4F). This implied that *Ser* and *mam*, the predicted target genes of dmiR-283 and key genes of the Notch pathway, were suppressed due to the accumulation of dmiR-283 expression. Thus, we predict that the age-dependent increase in sleep is attributed to the accumulation of brain dmiR-283, which suppresses *Ser* and *mam*, key genes of the Notch pathway, leading to a disorder of sleep behavior.

To further investigate the mechanisms underlying the reduced amplitude of sleep and activity in *Pdf* > mir-283^SP^ *Drosophila*, we intersected the predicted target genes of mircoT-CDS with 206 rhythmically expressed genes obtained from *Drosophila* brain transcriptome sequencing and found eight predicted genes for rhythmic expression (Figure 4G), of which *Pdp1* and *cwo* were the predominant clock genes [42]. It has been established that CWO, as a repressor of CLK-activated transcription, is an important transcription inhibitor of circadian rhythm amplitude [43], whereas the activator PDP1 regulates *Clk* transcription in conjunction with the repressor VRI [44,45]. We examined the brain levels of *Pdp1* and *cwo* in aging as well as LNvs knockdown dmiR-283, and found a significant age-dependent reduction in *cwo* expression as well as a dmiR-283-knockdown-induced reduction (Figure 4H), suggesting that a weakened rhythmic amplitude in aging or *Pdf* > mir-283^SP^ *Drosophila* may be associated with a decrease in *cwo*. In contrast, *Pdp1* expression increased and decreased in senescent mir-283^SP^/+ and *Pdf* > mir-283^SP^ *Drosophila* brains, respectively, which is inconsistent with the expected result of accumulation of dmiR-283-induced target gene degradation in senescent brains; therefore, we hypothesized that the regulation of circadian rhythm by *Pdp1* is not directly affected by dmiR-283 alterations.

### 2.5. Expression Levels of dmiR-283 in the Brain Play a Key Role in Response to Exercise Effects

**Figure 5:**
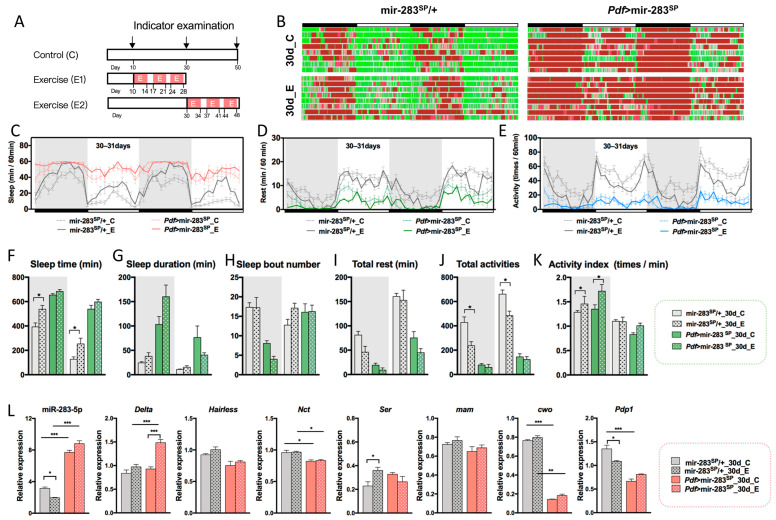
Effects of exercise initiated in youth on sleep–wake behavior and related gene expression in middle-aged *Drosophila*. (**A**) A simple diagram of the exercise program, with E1 indicating the start of exercise in youth to be tested in middle age and E2 indicating the start of exercise in middle age to be tested in old age. (**B**) Sleep–wake diagrams of mir-283^SP^/+ and *Pdf* > mir-283^SP^ in middle age for the non-exercise and E1 groups on 2 consecutive days, *n* = 25–32. (**C**–**E**) Sleep time per hour, rest time per hour, and the number of activities per hour for 2 consecutive days for age-matched non-exercise and E1 groups of mir-283^SP^/+ and *Pdf* > mir-283^SP^, respectively. (**F**–**K**) Total sleep time at night/day (Kruskal–Wallis test) (**F**), sleep duration at night/day (**G**), the number of sleep segments at night/day (**H**), total rest time at night/day (**I**), total activity numbers at night/day (**J**), and the number of activities per minute during wakefulness at night/day (**K**) of mir-283^SP^/+ and *Pdf* > mir-283^SP^ *Drosophila* in non-exercise and after E1 intervention. (**L**) The expression levels of dmiR-283-5p and its predicted target genes in the brain of *Drosophila* mir-283^SP^/+ and *Pdf* > mir-283^SP^ in non-exercise and after E1 intervention (one-way ANOVA test). * *p* < 0.05, ** *p* < 0.01, *** *p* < 0.001.

Our previous studies demonstrated that prolonged fatigue exercise increases sleep stability and improves sleep quality in aging *Drosophila* [46,47,48], but the regulatory mechanisms remain unclear. Based on the aging change characteristics of sleep–wake behavior, two exercise protocols were designed in this study: exercise starting from young adulthood to middle age (E1), and exercise starting from middle age to old age (E2) (Figure 5A), which aimed to investigate the effect of endurance exercise starting at different ages on age-dependent sleep–wake behavior decline.

In the E1 exercise protocol, we found that endurance exercise increased sleep time in mir-283^SP^/+ flies (Figure 5C,F). Although activity behavior decreased (Figure 5E,J), the activity frequency increased upon awakening (Figure 5K), as the activity index (the number of activities per minute) was a better indicator of health in the flies [22,23,48], indicating that exercise improved health in middle-aged *Drosophila*. JTK_CYCLE and CircaCompare analyses indicated that endurance exercise ameliorated age-dependent sleep cycle lengthening, allowing mir-283^SP^/+ *Drosophila* to still maintain a 12 h circadian oscillation with an increased rhythmic amplitude of sleep, activity, and rest (Figure 5C–E, Table S3), suggesting that endurance exercise initiated in youth maintains a relatively stable sleep–wake rhythm output. The expression of dmiR-283 was down-regulated after exercise in mir-283^SP^/+ brains, with a trend toward the increased expression of its predicted target genes *Ser* and *mam* (Figure 5L). Although the change in *cwo* was not significant, we found that exercise significantly reduced the accumulation of *Pdp1* expression in aging brains (Figure 5L), predicting that the improvement of circadian rhythm by endurance exercise may be related to exercise, suppressing the overexpression of *Pdp1* in the aging brain.

**Figure 6:**
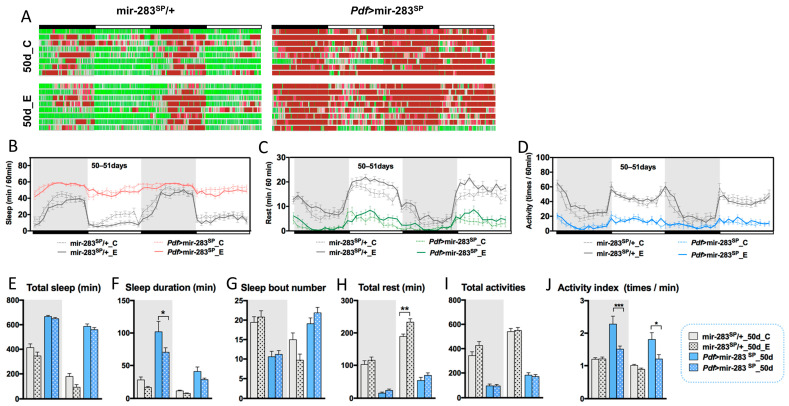
Effects of exercise initiated in midlife on sleep–wake behavior in aged *Drosophila*. (**A**) Sleep–wake diagrams for the non-exercise and E2 groups of elderly mir-283^SP^/+ and *Pdf* > mir-283^SP^ for 2 consecutive days, *n* = 22–32. (**B**–**D**) Sleep time per hour, rest time per hour, and the number of activities per hour for 2 consecutive days for non-exercise and E2 groups of mir-283^SP^/+ and *Pdf* > mir-283^SP^ elderly *Drosophila*. (**E**–**J**) Total sleep time at night/day (Kruskal–Wallis test) (**E**), sleep duration at night/day (**F**), the number of sleep segments at night/day (**G**), total rest time at night/day (**H**), total activity numbers at night/day (**I**), and the number of activities per minute during wakefulness at night/day (**J**) in non-exercise and E2 intervention of mir-283^SP^/+ and *Pdf* > mir-283^SP^ *Drosophila*. * *p* < 0.05, ** *p* < 0.01, *** *p* < 0.001.

*Pdf* > mir-283^SP^ flies appeared to be insensitive to exercise, with no significant changes in circadian rhythm amplitude and phase, except for a significant increase and decrease in the mesor of sleep and rest rhythms after exercise (Table S3). In addition, endurance exercise, while increasing the frequency of night-time activity in *Pdf* > mir-283^SP^ *Drosophila*, had no significant effect on most sleep–wake behaviors and related gene expression (Figure 5F–L), indicating that the overexpression of brain dmiR-283 may affect the ameliorative effect of exercise on the aging-related sleep–wake behavioral decline. The speculation was further validated by endurance exercise starting at mid-age (E2). Brain dmiR-283 expression was significantly higher in mir-283^SP^/+ *Drosophila* at 30d (Figure 4A), and the onset of endurance exercise at midlife was only effective for the maintenance of activity rhythms (Table S3), with no significant changes in other sleep–wake behaviors. The expression of dmiR-283 in the brain was increased 7.7-fold in *Pdf* > mir-283^SP^ middle-aged *Drosophila* compared with young mir-283^SP^/+ flies (Figure 4A), and sleep–wake behavior after endurance exercise (E2) showed negative effects such as decreased nocturnal sleep stability (Figure 6F) and reduced day/night activity frequency (Figure 6I,J), which further suggests that overexpression of dmiR-283 in the brain causes exercise to play a negative role.

## 3. Discussion

In recent years, an expanding number of studies have found that miRNA-mediated post-transcriptional regulation plays an important role in the generation and maintenance of biological clocks in *Drosophila* and mammals, but whether aging-related circadian rhythm disorders are associated with age-dependent alterations in miRNA expression remains unreported. In our study, we found that flies developed diminished sleep–wake rhythm amplitudes and decreased mesor in middle age, which further intensified in old age, and also displayed age-dependent changes in sleep, activity, and rest behaviors, which may be associated with the elevated expression of dmiR-283 in the brain. Increased dmiR-283 levels consistent with aging were detected in both mir-283^OE^/+ and *Pdf* > mir-283^SP^ young *Drosophila* brains, and sleep–wake behavior tended to change with aging, which evidenced that brain dmiR-283 is a key miRNA regulating sleep–wake behavior during aging.

Notably, we observed longer daytime sleep (comparable to nighttime sleep duration) in the 10d flies of *w^1118^*, as well as differences in sleep structure (sleep time, number of sleep episodes, and sleep latency) between *w^1118^* and *Canton S* strains as observed by Vienne et al. [17]. This could be due to the *white* mutation that exacerbates progressive retinal degeneration in *Drosophila* [49], further affecting sleep structures. Therefore, whether the age-dependent changes we observed in *w^1118^* are applicable to other *Drosophila* strains or species remains to be further investigated.

**Figure 7:**
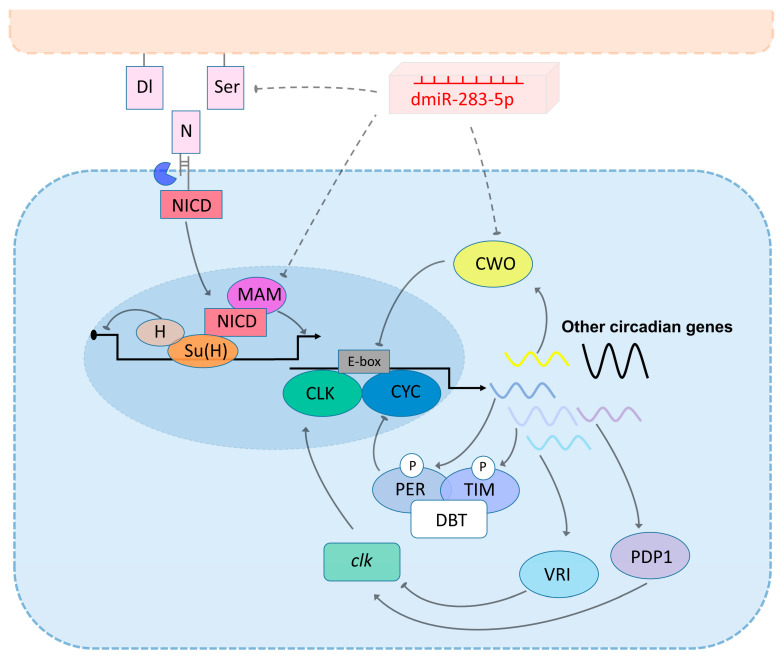
Potential mechanisms of dmiR-283-5p regulation of the Notch signaling pathway and circadian rhythm. The solid line with arrows indicates the facilitatory action; the solid line with ellipses indicates the inhibitory role; the dashed line with ellipses indicates the potential inhibitory effect of dmiR-283-5p on the Notch signaling pathway and circadian clock regulation.

Bioinformatic predictions revealed that dmiR-283 is mainly enriched in the Notch pathway, a ligand–receptor signaling pathway for the transmembrane transmission of information between cells, which is highly conserved evolutionarily [50,51]. In *Drosophila*, the Notch signaling pathway is activated by binding of the transmembrane receptor *Notch* (*N*) to the transmembrane ligand *Delta* (*Dl*) or *Serrate* (*Ser*) on adjacent cells, which hydrolyzes and releases the Notch intracellular structural domain (NICD). NICD translocates into the nucleus to interact with *Su* (*H*) and *mam* to form a transcriptional complex that activates downstream target gene transcription [51,52], and the repressor *Hairless* (*H*), which binds competitively to *Su* (*H*) and antagonizes the activation of the Notch signaling pathway [53] (Figure 7). Several studies have evidenced that Notch signaling plays an important role in sleep regulation in *C. elegans* and *Drosophila* [41,54,55]. *Notch* and *Delta* in the adult *Drosophila* brain can mediate interneuron–glial signaling through intercellular connections, and Notch signaling activation (overexpression of *Delta* and NICD, and the functional acquisition of *Notch* alleles) prevents the onset of sleep rebound after sleep deprivation and maintains sleep stability [54]. Our study reveals that dmiR-283 accumulates in aging and LNvs knockdown brains with a decreased expression of transmembrane ligand *Ser* and coactivator *mam* in its predicted target genes, predicting inhibition of the Notch signaling pathway, which may contribute to increased sleep. In mammals, Notch-related gene expression was downregulated in the hippocampus of sleep-deprived rats [56]; genome-wide association analysis has also shown a potential relationship between Notch signaling and insomnia [57], although the specific regulatory mechanisms between sleep/sleep-like behavior and Notch signaling need to be further explored.

Our study is the first to reveal that the expression of dmiR-283 exhibits 24 h circadian oscillations in young *Drosophila* brains and is predicted to be involved in the regulation of circadian rhythm amplitude as well. Of the predicted target genes, *cwo* and *Pdp1* constitute the core biological clock regulatory network. Expressed in rhythmically paced neurons, *cwo* serves as an important transcriptional repressor that regulates circadian rhythm amplitude, and its functional deficiency leads to a prolonged and weak autonomic activity rhythm in *Drosophila* [43,58,59], similar to the CLK–CYC transcriptional repression that prolongs or eliminates the circadian rhythm period [60]. Therefore, we hypothesized that the reduced circadian rhythm amplitude and prolonged cycles in aging and LNvs knockdown of dmiR-283 flies may be attributable to the degradation of *cwo* by the hyper-expression of dmiR-283 in the brain. Molecular studies have shown that the transcriptional activation of CWO is regulated by CLK–CYC, which, in addition to forming a self-regulatory negative feedback loop [61], synergistically represses the transcriptional activation of CLK target genes (*per*, *tim*, *vri*, and *Pdp1*) with PER [59] (Figure 7). A recent study also revealed that CWO promotes the transcriptional activation of CLK–CYC by repressing the negative regulator *Clock interacting protein circadian* [62]. In conclusion, the mechanisms of CWO in circadian rhythm regulation are becoming better defined, and our study confirmed that age-dependent declines in sleep–wake rhythm are associated with an accumulation of dmiR-283 in the brain. Additionally, a further suggestion is that dmiR-283 regulates the sleep–wake rhythm of aging or LNvs dmiR-283 knockdown *Drosophila* by targeting *cwo* for degradation.

A previous study proposed that the activation of vascular endothelial growth factor expression by the biological clock gene *Bmal1* crosstalks mechanistically with the Notch signaling pathway, regulating vascular sprouting and function [63]. Therefore, we hypothesized that the behavior of prolonged sleep and reduced rhythm of autonomous activity during aging in *Drosophila* is associated with an inhibited Notch signaling pathway and reduced expression of the core rhythm gene *cwo* in the brain, which are co-regulated negatively by the aging accumulation of dmiR-283 (Figure 7).

Exercise as a beneficial lifestyle has been identified as a positive contributor to healthy aging and longevity [2,64], not only by improving potential sleep disturbances in the young and old [65,66], but also by resetting the circadian biological clock [67,68,69]. Aerobic exercise, especially, has been shown to play a positive role in promoting brain health and healthy sleep patterns; thus, exercise has been proposed as a non-pharmacological intervention for patients with sleep disorders [70]. A study of young, healthy male subjects showed that 1 h of high-intensity exercise improved sleep quality by increasing slow-wave sleep [71]. Similarly, we found that endurance exercise starting in youth showed positive effects in healthy mir-283^SP^/+ flies, in terms of enhanced sleep–wake rhythm amplitude, stable sleep cycles, and increased activity frequency during waking. We propose that endurance exercise down-regulates dmiR-283 accumulated in the brain, thereby releasing the inhibition of the Notch signaling pathway by aging. Although the level of *cwo* did not change notably after exercise, we found that exercise significantly reduced the accumulation of *Pdp1* expression in aging brains, predicting that the improvement in circadian rhythm may be related to exercise suppressing the overexpression of *Pdp1* in the aging brain. *Pdp1*, a key activator of *Clk*, results in reduced CLK and PER expression and circadian rhythm dysfunctional behavior in the presence of either *Pdp1* mutations or a dysregulated *Pdp1*/*vri* ratio [44,72]. Our study further explored the exercise effects in the presence of high brain dmiR-283 expression (aging and LNvs knockdown of dmiR-283) and showed ineffective or negative sleep–wake behaviors, indicating a negative correlation between dmiR-283 expression levels and exercise benefits.

Aging is a major risk factor for most chronic diseases and functional disorders. Taking advantage of the short life span and the homologous molecular regulatory mechanisms of *Drosophila*, we found that increased sleep-like behaviors and reduced circadian rhythms during aging are associated with the accumulation of brain dmiR-283. Additionally, we reasonably speculate that the increased sleep behaviors probably occurred by suppressing the Notch-related pathway and weakening the circadian rhythm by depressing the expression of the core clock gene *cwo*. Thus, dmiR-283 may act as a co-regulator of the Notch signaling pathway and biological clock core genes to promote the aging process in the brain. Endurance exercise initiated in youth reduces brain dmiR-283 levels, maintaining a solid circadian output in aging *Drosophila*. In contrast, exercise performed at a certain accumulation level of brain dmiR-283 displayed ineffective or negative outcomes, indicating that the affirmative results of exercise are dependent on dmiR-283 stable-expression.

## 4. Materials and Methods

### 4.1. Drosophila Strains, Crosses, and Rearing

Wild-type strains (*w^1118^*, BS3605), driver strains of pigment dispersing factor (*Pdf*)-expressing ventral lateral neurons (*LNvs*) in the brain (*Pdf*-Gal4, BS41286), mir-283 knockdown strains (mir-283^SP^, BS61415, *w* [*]; *P*{*y* [*+t7.7*] *w* [*+mC*] = *UAS-mCherry.mir-283.sponge.V2*}*attP40*; *P*{*y* [*+t7.7*] *w* [+*mC*] = *UAS-mCherry.mir-283.sponge.V2*}*attP2*), and mir-283 knockout strains (mir-283^KO^, BS58912, w [*] TI{TI}mir-283 [KO]) were purchased from the United States Bloomington *Drosophila* Stock Center. The overexpression strain of mir-283 (mir-283^OE^, BCF132#, the attP40 landing site (Chr.2 25C6) stock used for the fly embryo injection and transformation was y [1] M{vas-int.Dm}ZH-2A w [*]; P{CaryP}attP40) was purchased from the Core Facility of *Drosophila* Resource and Technology, CEMCS, CAS. The cardiac-specific driver strain (*Hand*-Gal4) was presented by the Center for Heart Development of Hunan Normal University. Virgin flies of the mir-283^SP^ strain that fledged within 8 h were collected and crossed with male strains *w^1118^* and *Pdf*-Gal4; correspondingly, the F1 generation virgin flies contained control group (mir-283^SP^/+) and knockdown group (*Pdf* > mir-283^SP^). For reducing the effect of genetic background on *Drosophila*, virgin flies of pure mir-283^OE^ and mir-283^KO^ strains, which fledged within 8 h, were collected and crossed with male strain *w^1118^*, and F1 generation virgin flies were overexpressed (mir-283^OE^/+) or mutated (mir-283^KO^/+) heterozygous flies, corresponding to the wild-type (+) allele, and *w^1118^* virgin flies of the same age served as a control. All flies were reared in standard yeast flour/cornmeal/soybean flour/sucrose/maltose/agar medium under conditions of 25 ± 1 °C, 60% humidity, and a 12 h light and 12 h dark environment. For the analysis under constant darkness (DD), newly collected virgin flies were first entrained in light–dark conditions for 3 days and then reared in a DD environment for 3 days for testing. The remaining Drosophila were reared under standard conditions of 12 h light:12 h dark.

We conducted experimental comparisons between different sexes at the beginning of the experiment, and the results showed that male flies slept more at night and during the day, showing stronger rhythmicity than females. However, in the induced climbing movement experiment, males showed poor performance in negative tending ground climbing ability and endurance, which may reduce the effect of induced movement. Therefore, we chose females with higher locomotor ability as the experimental subjects. In addition, flies were housed in groups during rearing and hybridization, and in single housing in the activity monitoring experiment.

### 4.2. Exercise Training Program

Based on the anti-gravity climbing characteristics of *Drosophila*, we developed a next-generation *Drosophila* climbing exercise device (Patent No. CN202021948877.0), which induced flies to climb upward along the test tube wall by setting an arbitrary flipping angle and dwell time. In this study, the motor speed was set to 0.45 r/s, and a 180° vertical flip was performed and then left for 5 s, during which most flies were able to actively crawl upward to the top of the test tube.

According to the optimal exercise protocol determined in a previous study [47], flies were induced to perform 2.5 h of climbing exercise per day for 5 days followed by 2 days of rest, and the exercise program lasted for a total of 3 weeks. There is a distinct sleep–wake rhythm in young *Drosophila*, with sleep time concentrated in the middle of the day/night period and high activity before and after lights on/off (Figure 1A); therefore, the exercise protocol was performed during the peak activity period before lights off (ZT9~ZT12), to avoid inducing sleep deprivation. The 10th (10d), 30th (30d), and 50th (50d) days of age in *Drosophila* correspond to the young, middle-aged, and old stages [73]. To investigate the effect of exercise initiated at different periods on sleep–wake behavior in *Drosophila*, two exercise protocols were designed, as shown in Figure 5A.

### 4.3. Life Expectancy Statistics

The number of dead flies was recorded from the first day after fledging and counted during ZT14-ZT15 until all flies were dead. Flies were transferred to fresh medium every 3 to 4 days during the lifespan count. Based on the total number of flies and the number of survivors, survival curves were plotted and the average lifespan was calculated.

### 4.4. Sleep–Wake Behavior Analysis

During ZT12~ZT15, 2 days before data collection, age-appropriate flies (8d, 28d, and 48d) were transferred individually into a 5 mm × 65 mm polycarbonate tube with standard culture medium at one end and a sponge plug at the other end. Under standard experimental conditions, activity data were monitored for 3 consecutive days using a *Drosophila* activity monitoring system (DAMS, TriKinetics, Waltham, MA, USA), where an infrared beam is emitted at the center of each glass activity tube and the number of passes through the infrared beam is automatically recorded as the flies move back and forth through the monitoring tube, with data collection intervals of 1 min. Data from the first day were discarded to avoid the effect of changes in the survival environment [74,75]. Data from days 2 to 3 (9~11d, 29~31d, and 49~51d) were used for sleep–wake behavior and circadian rhythm analysis.

Behavioral and electrophysiological studies showed that the continuous 5 min inactivity of *Drosophila* resembles the mammalian sleep state [76,77,78]. Based on this feature, we analyzed DAMS data using a custom JavaScript script and visualized sleep–wake behavior with ECharts, in which sleep behavior was shown in red with darker colors indicating higher sleep persistence. To further study the arousal characteristics of *Drosophila*, we divided arousal into two states, active and resting. Activity means the time to cross the infrared beam, shown in green; resting means the flies did not cross the infrared beam for 1–4 min and, at this time, the flies are not active, but also not asleep, shown in gray. Both resting and active behaviors belong to the awakening state (Drosophila Sleep-Wake Behavior Analysis System V1.0, Computer Software Copyright Registration No.: 2022SR1402794).

### 4.5. Circadian Rhythm Analysis

Cycle analysis and circadian rhythm significance analysis were performed using JTK_CYCLE in the metacycle package for hourly sleep time, hourly rest time, hourly activity, and continuous 24 h gene expression levels in the brain. The circadian rhythm amplitude (half of the difference between the peak and trough), phase (time corresponding to peak), and mesor (response rhythm-adjusted average) were analyzed differentially according to the CircaCompare program developed by Parsons et al. [79] to plot circadian rhythm fitting curves.

### 4.6. Tissue Sample Collection, Total RNA Extraction, and RT-qPCR

Adult *Drosophila* brains were obtained with reference to the method described by Williamson and Hiesinger [80]. To investigate the circadian expression levels of genes, *Drosophila* brains were collected at 4 h intervals from ZT4 to ZT24, and the other brain tissue sampling was performed from ZT8 to ZT12. According to the instruction manual, total RNA was extracted using RNAfast200 (Shanghai Feijie Biotech). The Mir-X miRNA First-Strand Synthesis Kit and PrimeScript™ RT reagent Kit with gDNA Eraser (Takara Biomedical Technology (Beijing) Co., Ltd., Beijing, China) were used for miRNA and mRNA reverse transcription reactions, respectively. Quantitative RT-PCR assays were performed using TB Green^®^ Premix Ex Taq™ II (Takara Biomedical Technology (Beijing) Co., Ltd., Beijing, China) on a CFX Connect^TM^ fluorescent quantitative PCR instrument (Bio-Rad Laboratories, Berkeley, CA, USA). The primer sequences are shown in Table 1.

### 4.7. Reactive Oxygen (ROS) Testing

Brains were stripped in oxygen-enriched hemolymph solution, and the fluorescent probe of Dihydroethidium (DHE, Beyotime Biotechnology Co., Shanghai, China) was diluted to 1:1000 with an artificial hemolymph according to the superoxide anion fluorescent probe instructions. After incubation for 30 min at room temperature in a shaker, the brains were washed with hemolymph solution to adequately remove DHE that had not entered the cells, and were imaged immediately using a fluorescent microscope; all images were collected based on the same parameters. Relative ROS levels were measured by quantification of the dye fluorescence using ImageJ 1.53a software.

### 4.8. Fluorescence Imaging

Brain DHE fluorescence was photographed using a *Leica* stereomicroscope (*Leica*, DVM6) to detect ROS levels. In addition, the mir-283^SP^ strain of *Drosophila* carries a red fluorescent marker gene that expresses the red fluorescent protein when specifically knocking down the LNvs dmiR-283 after crossing with *Pdf*-Gal4. The expression of the red fluorescent protein in the *Drosophila* brain was photographed using a *Leica* stereomicroscope to determine if the strain was successfully constructed.

### 4.9. Bioinformatics Analysis

The DIANA microT-CDS online tool (http://diana.imis.athena-innovation.gr/DianaTools/ accessed on 1 August 2022) was used to predict target genes for dme-miR-283-5p, whereas the DAVID Bioinformatics Resource (https://david.ncifcrf.gov/ accessed on 1 August 2022) was utilized to perform pathway enrichment analysis of the predicted target genes. Moreover, the predicted target genes were intersected with the rhythmically expressed genes obtained by transcriptome sequencing of the brain of *w^1118^* strain wild-type *Drosophila* [42] to identify potential transcription factors of dmiR-283 regulating the circadian rhythm.

### 4.10. Data Analysis and Processing

SPSS 22.0 was used for statistical analysis of the data. For data obeying normal distributions with uniform variance, an independent-samples *t*-test or one-way ANOVA was performed, followed by Tukey’s multiple comparisons to assess significance. Otherwise, independent-samples nonparametric tests with Kruskal–Wallis multiple comparisons were used. Graphs were generated in GraphPad Prism 7 and data were expressed as mean ± standard error *(M ± SEM)* with a significance level of *p < 0.05*.

## 5. Conclusions

In summary, the accumulation of dmiR-283 expression in the brain induced an age-dependent decline in sleep–wake behavior, and the core clock gene *cwo* and Notch signaling pathway may co-mediate this regulation. Furthermore, commencing endurance exercise in youth counteracts the increase in dmiR-283 in the aging brain, which ameliorates the deterioration of sleep–wake behavior during aging. Meanwhile, the stable expression of dmiR-283 is a guarantee that endurance exercise delays brain aging.

## 6. Patents

This study produced a Computer Software Copyright Registration entitled “Drosophila Sleep-Wake Behavior Analysis System V1.0” (No.: 2022SR1402794).

## Figures and Tables

**Table 1:** Primer sequence.

Gene	Forward Primer	Backward Primer
*U6 snRNA*	5′-TGGCCCCTGCGCAAGGATG-3′	——
dmiR-283-5p	5′-TCTCGAATAGCGTTGTGACTGA-3′	——
*rp49*	5′-CTAAGCTGTCGCACAAATGG-3′	5′-AACTTCTTGAATCCGGTGGG-3′
*cwo*	5′-GCCGTATCGAGAAGACGGAG-3′	5′-TCCATGTAGCCACTCCGGTA-3′
*pdp1*	5′-CGTCCCCAACACTGATCGAA-3′	5′-TGTGTTACCTTGAGGTCGGC-3′
*Delta*	5′-GGGTACCTTCTCGCTGATCG-3′	5′-GTCCAAATGAATCGTCGCGG-3′
*Hairless*	5′-TTGGTGGCGGTCTAAGTCAC-3′	5′-GACTCCGTTTTCCTCCAGCA-3′
*Nct*	5′-GAAAGTGCGCAACGTTTCCT-3′	5′-GGCCTTAAAGAGTGGGCAGT-3′
*Ser*	5′-CACAGCCACCGCGATTATTG-3′	5′-GCAATCGCGACCCTTGAATC-3′
*mam*	5′-CGATCTCGGCTCATTGGACA-3′	5′-AAGCCATCGAGGAAACTGGG-3′

## Data Availability

Not applicable.

## Supplementary Materials

The following supporting information can be downloaded at: https://www.mdpi.com/article/10.3390/ijms24044180/s1.
