# Supplementary material for: Stable Expression of dmiR-283 in the Brain Promises Positive Effects in Endurance Exercise on Sleep–Wake Behavior in Aging Drosophila
Source: Int J Mol Sci. 2023 Feb 20;24(4):4180. doi: 10.3390/ijms24044180 (PMC9966282; doi:10.3390/ijms24044180)
Supplement: Supplementary file 1 [file ijms-24-04180-s001.zip › Table S1.pdf]

Table S1. CircaCompare analysis of sleep–wake rhythms in young, middle-aged and old *Drosophila.*

	**Sleep time / h**				**Rest time / h**				**Number of activities / h**			
	**JTK.PER**	**mesor**[a]	**amplitude**	**phase**	**JTK.PER**	**mesor**[a]	**amplitude**	**phase**	**JTK.PER**	**mesor**[a]	**amplitude**	**phase**
10d	12 h	19.28	14.11	6.09	12 h	7.64	1.57	11.08	12 h	72.03	28.66	0.43
30d	24 h	20.22	5.37**	6.79	24 h	8.16	0.79	0.24	24 h	60.99***	11.73***	1.13
50d	24 h	36.25###&&&	2.08&&&	8.09	24 h	9.27	0.69	2.93	24 h	26.82###&&&	2.28#&&&	1.82

Note: [a] denotes circadian-adjusted mean. * indicates the difference between 30d and 10d; # indicates the difference between 50d and 30d; & indicates the difference between 50d and 10d. *, #, and & $p<0.05$; **, ##, and && $p<0.01$; ***, ###, and &&& $p<0.001$.
