# Supplementary material for: Stable Expression of dmiR-283 in the Brain Promises Positive Effects in Endurance Exercise on Sleep–Wake Behavior in Aging Drosophila
Source: Int J Mol Sci. 2023 Feb 20;24(4):4180. doi: 10.3390/ijms24044180 (PMC9966282; doi:10.3390/ijms24044180)
Supplement: Supplementary file 1 [file ijms-24-04180-s001.zip › Table S2.pdf]

Table S2. CircaCompare analysis of sleep–wake rhythm of *Drosophila* with dmiR-283 knockout and overexpression.

	Sleep time / h				Rest time / h				Number of activities / h			
	**JTK.PER**	**mesor[a]**	**amplitude**	**phase**	**JTK.PER**	**mesor[a]**	**amplitude**	**phase**	**JTK.PER**	**mesor[a]**	**amplitude**	**phase**
w^{1118}	12	18.27	14.72	6.12	12	7.45	1.47	11.05	12	74.83	30.43	0.44
mir-283^{OE}/+	12	30.01***	21.65**	7.32***	12	8.09	5.68***	1.48***	12	39.59***	29.48	1.22***
mir-283^{KO}/+	12	14.08#	12.60	6.50	24	8.19	0.90	0.09	12	75.68	27.58	0.82

Note: [a] denotes circadian-adjusted mean. * indicates the difference between overexpression group and control group; # indicates the difference between knockout group and control group. *, #, and & p<0.05; **, ##, and && p<0.01; ***, ###, and &&& p<0.001.
