# Supplementary material for: Stable Expression of dmiR-283 in the Brain Promises Positive Effects in Endurance Exercise on Sleep–Wake Behavior in Aging Drosophila
Source: Int J Mol Sci. 2023 Feb 20;24(4):4180. doi: 10.3390/ijms24044180 (PMC9966282; doi:10.3390/ijms24044180)
Supplement: Supplementary file 1 [file ijms-24-04180-s001.zip › Table S3.pdf]

Table S3. CircaCompare analysis of the effects of LNvs-specific knockdown of dmiR-283 and endurance exercise intervention on sleep–wake rhythms in aging *Drosophila*.

	Sleep time / h				Rest time / h				Number of activities / h			
	JTK.PER	mesor[a]	amplitude	phase	JTK.PER	mesor[a]	amplitude	phase	JTK.PER	mesor[a]	amplitude	phase
mir-283^{SP}/+_10d	12 h	24.88	17.14	6.78	12 h	10.74	4.47	0.96	12 h	40.62	22.25	0.77
mir-283^{SP}/+_30d_C	22 h	20.86	7.29	7.77	24 h	10.25	0.64	3.35	12 h	45.81	12.89	1.62
mir-283^{SP}/+_30d_E	12 h	32.18***	13.80*	7.91	23 h	8.56*	3.53*	2.40	12 h	30.25***	17.37*	1.69
mir-283^{SP}/+_50d_C	23 h	27.58	8.48	7.91	24 h	11.01	1.79	2.51	23 h	35.28	10.66	1.57
mir-283^{SP}/+_50d_E	22 h	22.18	8.21	8.51	24 h	13.46*	2.05	3.53	12 h	37.23	10.48	2.03
Pdf> mir-283^{SP}_10d	12 h	43.35###	10.39#	7.28	23 h	5.34###	3.36	1.26	12 h	19.24###	12.02##	1.37
Pdf > mir-283^{SP}_30d_C	23 h	49.53###	6.14	7.95	23 h	4.06###	2.09	2.15	12 h	9.51###	6.29##	1.73
Pdf > mir-283^{SP}_30d_E	23 h	52.35*	3.58	8.66	23 h	2.72*	1.31	2.76	23 h	8.36	3.79	2.39
Pdf > mir-283^{SP}_50d_C	23 h	53.32###	1.98&	8.46	23 h	2.56###	0.73	2.56	23 h	10.33###	1.85##	2.21
Pdf > mir-283^{SP}_50d_E	22 h	50.81*	2.52	8.88	23 h	3.74**	1.11	3.32	22 h	10.59	2.54	2.50

Note: [a] denotes circadian rhythm-adjusted mean. # indicates LNvs-specific knockdown of dmiR-283 flies compared with age-matched control flies; * indicates non-exercise group from the same genetic background compared with the exercise group. *, #, and & p<0.05, **, ##, and && p<0.01, ***, ###, and &&& p<0.001.
